# Growing plants in fluctuating environments: why bother?

**DOI:** 10.1093/jxb/ery312

**Published:** 2018-09-14

**Authors:** Shizue Matsubara

**Affiliations:** IBG-2: Plant Sciences, Forschungszentrum Jülich, D-52425 Jülich, Germany

**Keywords:** Circadian clock, fluctuating light, growth, metabolism, photosynthesis, variable temperature

## Abstract

This article comments on:

Annunziata MG, Apelt F, Carillo P, Krause U, Feil R, Koehl K, Lunn JE, Stitt M. 2018. Response of Arabidopsis primary metabolism and circadian clock to low night temperature in a natural light environment. Journal of Experimental Botany 69, 4881–4895.

**Light and temperature are highly dynamic environmental factors, but plant science research frequently ignores this variation to allow reproducible growth-chamber experiments. Using Arabidopsis and looking at changes over a 24 h period, Annunziata *et al.* (2018) scrutinized changes in metabolites and gene expression of circadian oscillator components under naturally varying light and temperature. They show that fluctuations modify systems organization and outputs, and propose that diel metabolism is adapted to covariation of light and temperature.**

Climate chambers have become indispensable for plant science research. We benefit greatly from the possibility of controlling environmental variables such as photoperiod, light intensity, air temperature and relative air humidity, and this enables the study of plant responses to specific environments and repeated plant cultivation and experiments under defined conditions. Typically, conditions inside climate chambers are kept constant except when the illumination is switched on/off to simulate day/night. Whilst such quasi-binary environments are relatively easy to reproduce and use for growing plants, we know that they are very different from field conditions in which environmental fluctuations are the rule rather than the exception (Box 1). So what changes in plants when we grow them in fluctuating versus constant environments?

**Box 1. Diel variations in light, temperature and gene expression of the plant circadian oscillator**

The diurnal time course of light intensity exhibits a sine curve under a clear sky (upper panel, yellow area on the left). Photosynthetically active photon flux density (PPFD) peaks around noon, reaching >1800 μmol m^–2^ s^–1^. In this example (all data from June 2017, near Bonn, Germany; photoperiod ~16.5 h) air temperature (solid red line) increases by >10 °C in 4–5 h from dawn and continues to increase until late afternoon, followed by a rapid decline also by >10 °C till dusk. The daily mean temperature is 21 °C with a diurnal variation of 19 °C. The orange areas at the bottom and the broken red line represent a square-wave light regime in a climate chamber at PPFD 150 μmol m^–2^ s^–1^ and 22 °C/18 °C day/night (daily mean 20 °C). While the conditions in the climate chamber stay the same, light and temperature vary from day to day in the field. With increasing cloud cover (in the middle and on the right) the daily light integral (DLI) and daily maximum PPFD as well as the daily mean and maximum temperature decrease while the daily minimum temperature increases. Cloud movement causes fluctuations of irradiance. In reality light fluctuation is far more dynamic than shown in this figure which is based on 30-min average data.

The panel at bottom left depicts sequential gene expression of circadian oscillator components over 24 h. The x-axis matches the time axis of the first diel cycle in the upper panel. Different colours of the lines correspond to the colours of oscillator components shown on the right. For clarity, expression curves are drawn with equal peak height and width. Their peak times are according to the observations by Annunziata *et al.* (2018) under a sinusoidal light regime (photoperiod 12 h; max. PPFD 465 μmol m^–2^ s^–1^; DLI 12 mol m^–2^ d^–1^) at 21–22 °C/20 °C. At dawn CIRCADIAN CLOCK ASSOCIATED 1 (CCA1) and LONG ELONGATED HYPOCOTYL (LHY) suppress expression of all the other components, namely *PSEUDO RESPONSE REGULATOR* (*PRR*) genes (*PRR9*, *PRR7*, *PRR5* and *PRR1* alias *TIMING OF CAB EXPRESSION 1*, *TOC1*), *GIGANTEA* (*GI*) and evening complex (*EC*) genes including *LUX ARRHYTHMO*, *EARLY FLOWERING 3* (*ELF3*) and *ELF4* (not shown). Sequential expression of PRRs then represses *CCA1* and *LHY* from noon to evening and TOC1 represses all the others but *ELF3* in the evening. Later EC maintains the repression of *PRR9*, *PRR7* and *GI*. Additionally, CCA1 and LHY as well as the different PRRs repress each other’s expression. The simplified illustration and description of the negative transcriptional feedback loops of the plant circadian oscillator are both according to Nohales and Kay (2016).

**Figure:**
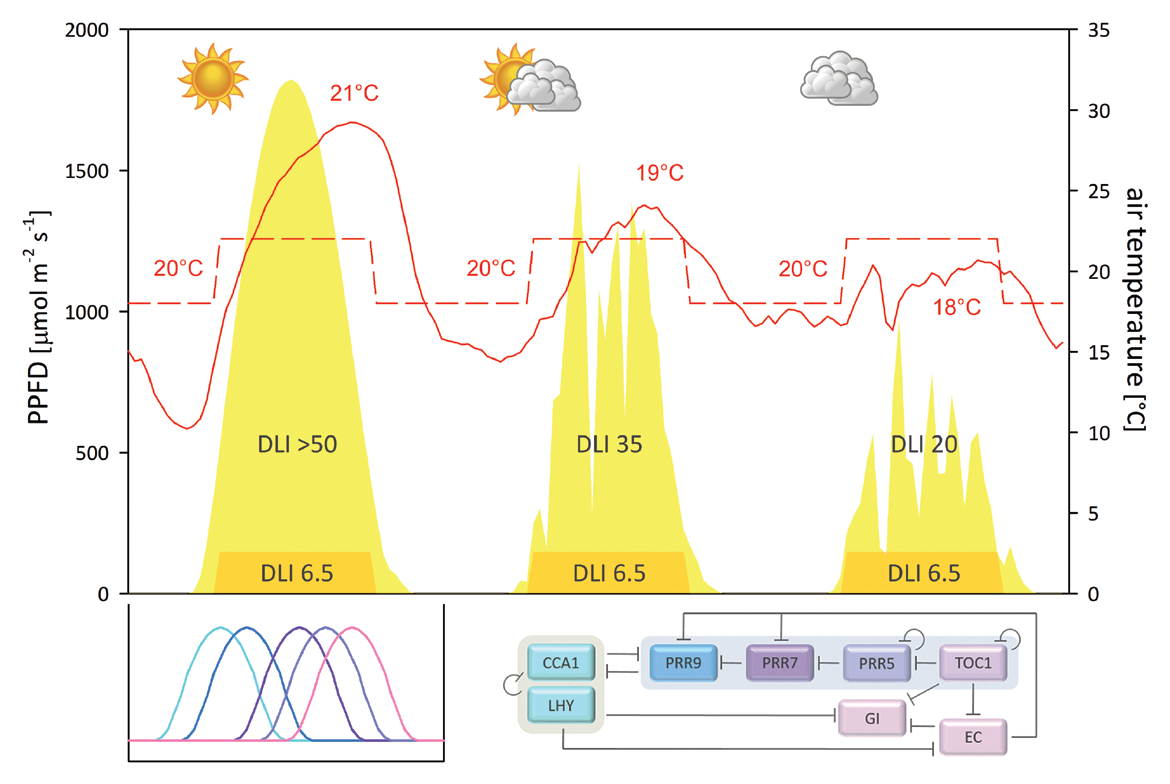

## Plants growing in fluctuating light

In a range of species leaf dry mass per unit leaf area (LMA), a key functional trait related to photosynthesis, growth and life history strategy of leaves (Wright *et al.*, 2004), increases in the field compared to controlled environments, presumably because the daily light integral (DLI) is much higher and temperature much lower in the field (Poorter *et al.*, 2016). For a given combination of DLI and temperature, however, LMA is influenced by temporal variation in irradiance. LMA declines in Arabidopsis under fluctuating light compared to 12 h/12 h light/dark (square-wave light) conditions (Vialet-Chabrand *et al.*, 2017), suggesting a decrease in effective DLI under fluctuating light. Indeed, upon sudden increase or decrease in irradiance photosynthetic light use efficiency is limited by enzyme activation in Calvin–Benson cycle and CO_2_ diffusion (Pearcy, 1990; Kaiser *et al.*, 2018) or slow relaxation of energy dissipation (Zhu *et al.*, 2004; Kromdijk *et al.*, 2016), respectively. Daily carbon gain and growth diminish in fluctuating light compared to square-wave light of the same DLI (Leakey *et al.*, 2002; Alter *et al.*, 2012; Vialet-Chabrand *et al.*, 2017) although species may differ in their responses (Watling *et al.*, 1997).

Concomitant with LMA and growth reduction, plants in fluctuating light also show other responses that are not found in square-wave light. At low DLI (3.5 or 5 mol m^–2^ d^–1^) square-wave light promotes light energy utilization and leaf expansion in Arabidopsis whereas fluctuating light, which comprises short lightflecks triggered under low-intensity background light (situations for inner-canopy leaves and understorey plants), enhances photoprotective energy dissipation and scavenging of reactive oxygen species (Alter *et al.*, 2012). In contrast, conditions mimicking natural light fluctuation at higher DLI (10 or 20 mol m^–2^ d^–1^; situations for sun-exposed leaves) were shown to increase the maximum CO_2_ assimilation per unit leaf mass compared to the corresponding square-wave light. Hence Arabidopsis leaves maintain the maximum CO_2_ assimilation per unit area as they become thinner and the total leaf area declines under fluctuating light (Vialet-Chabrand *et al.*, 2017).

Recently Annunziata *et al.* (2017) reported remarkable changes in leaf metabolism occurring under natural fluctuating light. Compared to square-wave or sinusoidal light regimes, natural illumination in otherwise similar environmental conditions attenuated connectivity between C and N metabolism in Arabidopsis. Natural light varies from day to day depending on the weather whereas the same light programme repeats every day in controlled environments (Box 1). Both within- and between-day fluctuations of irradiance thus affect metabolic profiles of plants under natural illumination. Notably, metabolic and photosynthetic properties were marginally altered in Arabidopsis when different light sources (fluorescent tubes versus light-emitting diodes) and regimes (square-wave versus sinusoidal) were compared under controlled environments (Annunziata *et al.*, 2017; Köhl *et al.*, 2017). These findings further underpin a major impact of light fluctuation.

## Plants growing in fluctuating light and temperature

Deviation from reality is incidental not only to light but also to temperature conditions in controlled environments (Box 1). While the majority of experiments in climate chambers are conducted at ~20 °C and with day/night variations (if any) of <6 °C, the daily mean temperature in temperate zones is ~10 °C in spring and 15–20 °C in summer, but with diurnal variations of 12–13 °C (Poorter *et al.*, 2016). Getting one more step closer to the field, Annunziata *et al.* (2018) extended the study to include temperature fluctuation. The authors compared sinusoidal light and natural illumination at 20–22 °C (Annunziata *et al.*, 2017) versus natural illumination at variable temperature. The daily mean temperature under the latter conditions ranged over 14–22 °C during the experiment, with diurnal variations of 4–19 °C (on average 10 °C). The DLI on harvest day was 12–14 mol m^–2^ d^–1^ in all conditions.

The picture emerging from their metabolome analysis (Annunziata *et al.*, 2018) illustrates how light and temperature fluctuations modify systems organization and outputs. Diel (over a 24 h period) C and N metabolism are strongly and positively correlated in Arabidopsis leaves under sinusoidal light at constant temperature. At the same DLI and temperature, fluctuating light dramatically weakens this correlation by partially uncoupling C and N metabolism and suppressing the accumulation of major amino acids and some organic acids. Combined variation of light and temperature, without further weakening or restoring the metabolic connectivity, brings about changes in specific compounds, such as retention of starch and sugars at dawn or marked increase in anthocyanins, amino acids (including proline) and proteins.

The variable responses of amino acids and organic acids were interpreted as an indication of weak buffering of the related pathways against environmental fluctuations (Annunziata *et al.*, 2018). One may also wonder whether flexible adjustment of metabolic connectivity is part of a system-level buffer to cope with fluctuations; it was not selected against during evolution (in the field). Seeing the changes in these compounds under variable light and temperature, the authors point to a striking resemblance to low temperature acclimation (Strand *et al.*, 1999; Stitt and Hurry, 2002). Accordingly, low night temperature slows down nocturnal starch mobilization and growth while up-regulating gene expression of ribosomal proteins (Usadel *et al.*, 2008) to enhance translation. The resulting increase in leaf protein content may augment photosynthesis in fluctuating light and promote growth during the day (Annunziata *et al.*, 2018). This hypothesis, which has yet to be tested, provides a possible explanation for apparent lack of growth penalty under natural light and variable temperature conditions.

## The circadian clock in natural fluctuating environments

In parallel with the metabolic reprogramming, Annunziata *et al.* (2018) identified peculiar changes in gene expression of circadian oscillator components under fluctuating environments. Particularly, activation of dawn-phased MYB transcription factor genes *CIRCADIAN CLOCK ASSOCIATED 1* (*CCA1*) and *LONG ELONGATED HYPOCOTYL* (*LHY*) was delayed by ~2 h under natural illumination and variable temperature, which was accompanied by increased or earlier expression of *PSEUDORESPONSE REGULATOR 9* (*PRR9*), *PRR7* and *GIGANTEA* (*GI*) in the morning. The central oscillator of the plant circadian clock is composed of interlocked transcriptional and translational feedback loops (Box 1). Whilst the period length of circadian-regulated gene expression is robust against fluctuations in the field (Nagano *et al.*, 2012), cool nights seem to fine-tune the timing and amplitude of specific clock gene expression (Annunziata *et al.*, 2018). What could such fine-tuning be good for?

Circadian clocks allow organisms to synchronize physiological and developmental processes, including growth, metabolism, stress responses and flowering, with diel and seasonal cycles of their environment (Nohales and Kay, 2016). In the case of low temperature acclimation, CCA1 and LHY are in charge of circadian-gated induction of *C-REPEAT BINDING FACTOR 1* (*CBF1*), *CBF2* and *CBF3* encoding AP2/ERF family transcription factors which regulate chilling/freezing tolerance (Dong *et al.*, 2011). CCA1 and LHY also act as pacemakers of nocturnal starch mobilization, preventing premature starch exhaustion before dawn (Graf *et al.*, 2010). Their importance is manifested in the Arabidopsis *cca1lhy* (or *lhycca1*) mutant and CCA1-overexpressor. These plants suffer from starch exhaustion and high starch retention, respectively, both resulting in nocturnal growth inhibition (Yazdanbakhsh *et al.*, 2011; Ruts *et al.*, 2012; Apelt *et al.*, 2017), although this does not apply to hypocotyl elongation, which is controlled by the evening complex (EC) and light (Nozue *et al.*, 2007; Nusinow *et al.*, 2011).

Does slow activation of *CCA1* and *LHY* following a cool night prolong starch mobilization after dawn and thereby redirect C to sucrose and amino acids (Annunziata *et al.*, 2018)? Does the higher or earlier expression of *PRR9*, *PRR7* and *GI* (Annunziata *et al.*, 2018) also modulate circadian-regulated processes as the temperature rises quickly in the morning (Box 1)? Interestingly, these five components (CCA1, LHY, PRR9, PRR7 and GI) are implicated in temperature compensation of clock oscillation (Nohales and Kay, 2016), which would gain more importance under fluctuating temperature. The study by Annunziata *et al.* (2018) raises many questions and inspires further investigation of the roles of the plant circadian clock in natural fluctuating environments.

## Future perspectives

More realistic though the results may be, experiments under natural irradiance and temperature have the significant drawback of low reproducibility. Moreover, preceding weather can influence the observations in present-day conditions, as noted by Annunziata et al. (2018), thus complicating data analysis and interpretation. Whilst such previous history effects are not always a problem (and can also be a fascinating subject of research), it is difficult to test hypotheses and validate models if we are not able to repeat the experiments. The good news: with the latest advances in climate chamber technology it is becoming possible to simulate light and temperature fluctuations in controlled environments. While factors like wind, rain and rhizosphere are not considered, light and temperature simulation will help us identify mechanisms and components which facilitate plant growth and confer resilience in fluctuating environments. The first examples demonstrating the power and utility of this approach for photosynthetic phenotyping have been reported in recent years (Cruz *et al.*, 2016; Rungrat *et al.*, 2016).

Plants can anticipate recurrent changes in their environment, above all in light and temperature. Plants are fairly safe in expecting a particular temporal pattern of light and temperature covariation in the field: lower temperature and dim or no light from dusk to dawn followed by a parallel increase in both in the morning. Even stochastic fluctuations of light, which are caused by clouds, wind and neighbouring plants, can be expected to occur during the daytime, in much the same way as the operation of photosynthesis. Given that seasonal changes in mean temperature and photoperiod or DLI are correlated in all major climate regions but in distinct ways (Hut *et al.*, 2013; Poorter *et al.*, 2016), and since photoperiod is known to be measured by the clock, anticipation and detection of the temperature–photoperiod relationship could be another important function of the clock in plant adaptation and acclimation to different environments. As we try to mimic natural light and temperature conditions in climate chambers, we may more often come across the ‘biological clockwork’ in the act of preparing for and responding to ever-fluctuating environments.
